# EUCALYPT: efficient tree reconciliation enumerator

**DOI:** 10.1186/s13015-014-0031-3

**Published:** 2015-01-23

**Authors:** Beatrice Donati, Christian Baudet, Blerina Sinaimeri, Pierluigi Crescenzi, Marie-France Sagot

**Affiliations:** Inria Grenoble - Rhône-Alpes; Inovallée 655, avenue de l’Europe, Montbonnot, Saint Ismier cedex, 38334 France; Université de Lyon, F-69000, Lyon; Université Lyon 1; CNRS, UMR5558; 43 Boulevard du 11 Novembre 1918, Villeurbanne cedex, 69622 France; Università di Firenze, Dipartimento di Ingegneria dell’Informazione; Via Santa Marta, 3, Firenze, 50139 Italy

**Keywords:** Cophylogeny, Reconciliation, Enumeration algorithm, Polynomial delay, Host-parasite systems

## Abstract

**Background:**

Phylogenetic tree reconciliation is the approach of choice for investigating the coevolution of sets of organisms such as hosts and parasites. It consists in a mapping between the parasite tree and the host tree using event-based maximum parsimony. Given a cost model for the events, many optimal reconciliations are however possible. Any further biological interpretation of them must therefore take this into account, making the capacity to enumerate all optimal solutions a crucial point. Only two algorithms currently exist that attempt such enumeration; in one case not all possible solutions are produced while in the other not all cost vectors are currently handled. The objective of this paper is two-fold. The first is to fill this gap, and the second is to test whether the number of solutions generally observed can be an issue in terms of interpretation.

**Results:**

We present a polynomial-delay algorithm for enumerating all optimal reconciliations. We show that in general many solutions exist. We give an example where, for two pairs of host-parasite trees having each less than 41 leaves, the number of solutions is 5120, even when only time-feasible ones are kept. To facilitate their interpretation, those solutions are also classified in terms of how many of each event they contain. The number of different classes of solutions may thus be notably smaller than the number of solutions, yet they may remain high enough, in particular for the cases where losses have cost 0. In fact, depending on the cost vector, both numbers of solutions and of classes thereof may increase considerably. To further deal with this problem, we introduce and analyse a restricted version where host switches are allowed to happen only between species that are within some fixed distance along the host tree. This restriction allows us to reduce the number of time-feasible solutions while preserving the same optimal cost, as well as to find time-feasible solutions with a cost close to the optimal in the cases where no time-feasible solution is found.

**Conclusions:**

We present Eucalypt, a polynomial-delay algorithm for enumerating all optimal reconciliations which is freely available at http://eucalypt.gforge.inria.fr/.

**Electronic supplementary material:**

The online version of this article (doi:10.1186/s13015-014-0031-3) contains supplementary material, which is available to authorized users.

## Background

Phylogenetic tree reconciliation has been the approach of choice for investigating the coevolution of sets of organisms such as hosts and parasites [1-3]. Besides the increasingly important role that reconciliation methods are likely to play in the study of coevolution, they have the advantage of being applicable to different types of data. For instance, they are extensively used for analysing the associations between genes and species [4-6], and between species and geological history [7]. The similarity between all three classes of problems was pointed out by Page already in 1994 [8] and further considered in [9,10]. More recently, a unique generalised formal model appeared in [11]. In this paper, we focus on the host/parasite associations but we want to call attention to the fact that, due to the similarity of the models, our algorithm can be straightforwardly applied to the other problems as well.

Reconciliation is modelled as a tree mapping problem – of the parasite tree onto the host one – with constraints. During the mapping process, four types of events are considered [1,12]. These are: cospeciation (this happens when both host and parasite speciate), duplication (when the parasite speciates but not the host, both new parasite species remaining associated with the host), loss (when the host speciates but not the parasite, leading to the loss of the parasite in one of the two new host species), and host switch (when the parasite speciates, one species remaining with its current host while the other switches, that is jumps to another). In the context of gene-species associations, this model is known as the *DTL* (for “Duplication, Transfer, and Loss”) model for the reconciliation problem and has been extensively studied (see, for example, [4-6,13,14]).

In the reconciliation problem, we are given a host tree *H*, a parasite tree *P*, and a mapping of the leaves of *P* to the leaves of *H* which reflects current knowledge on which existing parasites inhabit which hosts. By assigning a cost to each of the four types of events, we can obtain a parsimonious solution (or simply a reconciliation) according to the *DTL* model which minimises the total cost of the mapping. Additionally, if timing information is available, i.e. if we happen to know the order in which speciation events occurred in the host phylogeny, then any proposed reconciliation must also respect the temporal constraints imposed by the available timing information. In this case, the reconciliation problem can easily be solved using dynamic programming, in time polynomial in the size of the trees [15]. However, timing information may not be available or may be insufficiently reliable to be used with enough confidence. In such case, the reconciliation problem is NP-hard [5,6,16]. A number of algorithms have been developed that allow for solutions that are biologically unfeasible, that is for solutions where some of the switches induce a contradictory timing ordering of the internal vertices of the host tree [13,14,17,18]. In this case, the algorithms are able to generate optimal solutions in polynomial time without guaranteeing the time feasibility constraint.

This is the situation we address in this paper. We treat the reconciliation problem in the absence of any timing information, i.e. the two phylogenetic trees are provided undated. In this context, there are two main issues that must be taken into account in the reconciliation approach. First, providing a single optimal solution is not a good option as it can be biologically unfeasible (observe however that this is what the majority of the existing reconciliation algorithms do). Second, given a cost model for the events, an exponential number of optimal reconciliations is possible. Thus, even when restricting to time-feasible solutions, this number can remain huge. For these reasons, the capacity to enumerate all optimal solutions becomes a crucial point.

To the best of our knowledge, the reconciliation algorithms that try to deal with more than one optimal solution are the following: CORE-PA [19], MOWGLI [18], JANE 4 [16], NOTUNG [14], and RANGER-DTL [20]. However, MOWGLI assumes that the host and parasite trees are fully dated and computes just the number of optimal reconciliations without generating them. JANE 4 uses a heuristic based on a genetic algorithm for finding one or a number of solutions (not all and not necessarily of optimal cost). RANGER-DTL can handle both dated and undated trees and can compute the total number of optimal reconciliations. However, the currently available version of RANGER-DTL outputs only one optimal reconciliation. EUCALYPT and NOTUNG are the only publicly available algorithms that claim to generate all optimal reconciliations. However for most instances, CORE-PA enumerates only a proper subset of all optimal solutions (see the Results and discussion Section). NOTUNG was designed for a more general event model that includes duplications, losses, transfers, and incomplete lineage sorting (ILS). In particular, the *DTL* model is a special case when the species tree is binary. However, the algorithm imposes some restrictions on the cost values. Indeed, the cost of a cospeciation is always assumed to be equal to 0 and the cost of a loss positive.

We provide an algorithm that, given a cost model for the events, efficiently generates all the optimal solutions for the reconciliation problem. It is also possible for the algorithm to generate only optimal reconciliations that are time-feasible. EUCALYPT requires no assumption to be made concerning the cost values: it thus allows negative ones while cospeciation and loss may have any arbitrary cost. In addition, the algorithm can efficiently handle distance-bounded host switches, i.e. cases where the host switches are allowed to happen only between species that are within some fixed distance along the host tree. Observe that this is not an artificial requirement. The significance of a host switch distance has already been pointed out in several studies [21,22]. Indeed, if parasites switched only between closely related hosts, this would lead to a higher degree of congruence between the parasite and host trees. When this information is available, it should thus be taken into account in the reconciliation process. Moreover, it can happen that for some datasets and cost vectors, there is no optimal time-feasible solution. One way to overcome this problem can be by varying the length of the farthest allowed switch until at least one time-feasible solution is obtained. On the contrary, in the case where the number of optimal time-feasible reconciliations is high, one can decrease their number by selecting a subset of them. This can be done by decreasing the value of the maximum allowed distance of a switch while maintaining the same optimal total cost. Finally, the complexity of the bounded-switch problem remains open, and it could be that this constraint makes the reconciliation problem solvable in polynomial time, which in turn is of both theoretical and practical interest.

We show that the algorithm we developed and describe in this paper, EUCALYPT (for “EnUmerator of Coevolutionary Associations in PoLYnomial-Time delay” with one switch, of “P” before “LY”), loses no optimal solution, and is able to list all of them in linear-time delay: the time required for getting from one solution to the next one is indeed *O*(*m*) for *m* the number of vertices in the parasite tree, while finding the first solution requires *O*(*n*^3^*m*) time for *n* the number of vertices in the host tree. The space complexity for the whole enumeration process is also *O*(*n*^3^*m*).

We applied EUCALYPT to a number of host-parasite trees available in the literature, and to our own set of interest. We show that in general many optimal solutions exist. Indeed, as already noticed in other studies (see e.g. [20]) the number can sometimes be huge. We give an example where, for two pairs of host-parasite trees having each less than 41 leaves, the number of solutions is 5120, even when only time-feasible ones are kept. Depending on the cost vector, this may increase considerably (for the same example, to 4080384). EUCALYPT indeed comes with a procedure (in *O*(*n*^3^) time) for testing the time feasibility of a solution. The possibility to calculate the number of solutions without explicitly listing them all was also integrated in EUCALYPT. This has the same complexity as to enumerate a single solution. Finally, to facilitate interpreting the results even when a huge number of solutions is observed, the latter are classified in terms of the number of each event (cospeciation, duplication, loss, or host switch) that they contain. The number of different classes of solutions that must be examined further is often considerably smaller, but may remain high enough (for instance 275) depending on the cost vector.

## Implementation

### Model

A rooted phylogenetic tree is a leaf-labelled tree that models the evolution of a set of taxa from their most recent common ancestor (placed at the root). The internal vertices of the tree correspond to the speciation events. The tree is rooted so a direction is intrinsically assumed that corresponds to the direction of evolutionary time. Henceforth, by a phylogenetic tree *T*, we thus mean a rooted tree with labelled leaves and where the root has in-degree 0 and out-degree 2, the leaves have out-degree 0 and in-degree 1 and every other vertex has in-degree 1 and out-degree 2.

The model of host-parasite evolution we rely on in this paper is the event-based one presented by Tofigh *et al.* [6], and later further analysed by Bansal *et al.* [13]. Let *H*,*P* be the phylogenetic trees for the host and parasite species respectively. We define *ϕ* as a function from the leaves of *P* to the leaves of *H* that represents the association between currently living host species and parasites. Such association is an input of our algorithm, together with the trees themselves. In this model, we allow each parasite to be related to one and only one host, while a host can be related to zero, one, or more than one parasite.

**Figure 1:**
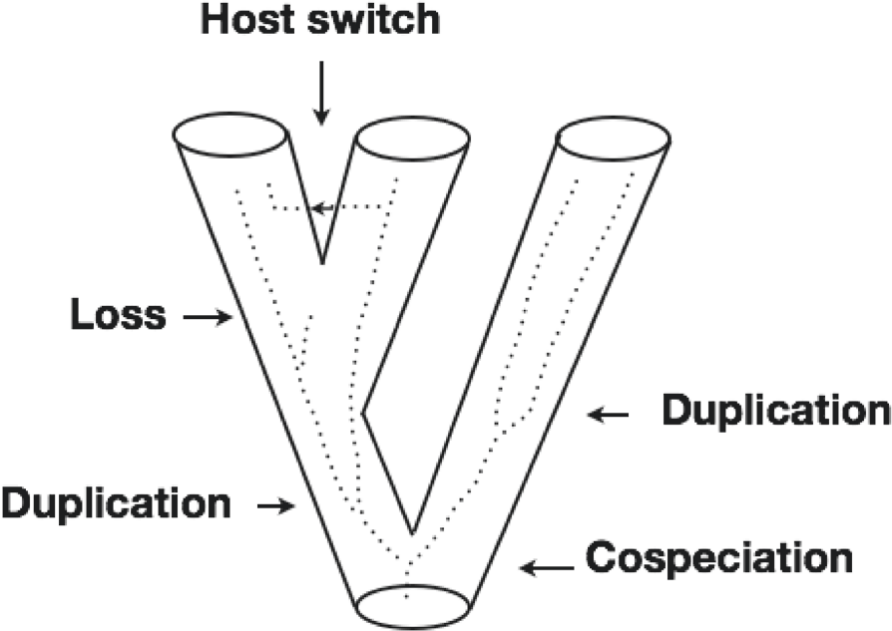
**Recoverable events for a coevolutionary reconstruction.** Schematic representation of cospeciation, duplication, host switch and loss events. The tube represents the host phylogenetic tree while the dotted line the one of the parasite.

In studying the coevolution of hosts and parasites, the following set of events are generally allowed to take place (see Figure 1): (a) cospeciation: the host and parasite speciate concurrently, (b) duplication: the parasite speciates independently from the host and both new species of parasites remain with the host, (c) loss: the host speciates but the parasite does not, and (d) host switch: the parasite speciates but one of the new parasite species switches (jumps) to another host.

A reconciliation is a function *γ* from the set of nodes of *P* to the nodes of *H* that extends the mapping of the leaves *ϕ*. Besides this, *γ* must satisfy some logical constraints as for example: (a) a node of *P* cannot be mapped in an ancestor of the mapping of its father; and (b) one of the two children of an internal node *v* of *P* must be mapped in the subtree of *H* rooted at *γ*(*v*), the image of *v* in *H*.

A reconciliation can be uniquely associated to a multi-set of events from cospeciation, duplication and switches. Indeed, for each node of the parasite tree, one can determine the event associated to that node by looking at the mappings of its children. For instance, a node *v* is associated with a cospeciation if its children are mapped in different subtrees rooted at the two children of *γ*(*v*). Finally, losses are identified by a multi-set containing all the vertices *h*∈*V*(*H*) that are in the path from the image of a vertex in *V*(*P*) and the image of one of its children. A detailed and formal description of the model can be found in Additional file 1.

The triple *S*=〈*H*,*P*,*γ*〉 is said to be a *scenario* or simply a reconciliation. Given a *vector* 〈*c*_*c*_,*c*_*d*_,*c*_*s*_,*c*_*l*_〉 of real values that correspond to the cost of each type of event, the optimal reconciliations are the ones that minimise the total cost.

Finally, some combinations of host switches can introduce an incompatibility due to the temporal constraints imposed by the host and parasite trees, as well as by the reconciliation itself. Determining whether a reconciliation is time-feasible can be done in polynomial time [14]. It is common to refer to a time-feasible (unfeasible) solution as acyclic (cyclic), and in this paper we will use these terms interchangeably.

*The All Most Parsimonious Reconciliation problem* (*All-MPR problem*) consists in generating all reconciliations of minimum cost. Finally, we consider the problem when the host switches are distance-bounded, *i.e.* there exists a bound on the maximum distance *k* along the host tree to which a parasite can jump. We call this the *k-bounded-All-MPR problem*. Clearly this is a generalisation in the sense that we trivially obtain the unbounded version by setting *k* equal to the longest path in the host tree.

We describe next the algorithm EUCALYPT that solves both problems in polynomial delay. As the set of optimal reconciliations produced may contain both cyclic and acyclic solutions, we also include an acyclicity test based on [14], for selecting only the time-feasible reconciliations.

### Algorithm

#### Finding one solution

In the same way as the algorithms which find one single reconciliation (possibly cyclic) of minimum cost, that is, which solve the so-called *DTL* problem [6,13,14], EUCALYPT uses a dynamic programming approach to find one or to enumerate all optimal reconciliations. In this approach, each (*p*,*h*) cell of the *m* by *n* dynamic programming matrix, let us denote it by *D*, contains a single (real or integer) number that represents the cost of an optimal (sub-)reconciliation mapping vertex *p* in the parasite tree to vertex *h* in the host tree. The matrix is filled following a post-order traversal of *P* and *H*. Bansal *et al.* provided an algorithm that finds the cost of one optimal reconciliation in time and space *O*(*nm*) [13]. We adapted the algorithm for solving the more general *k-bounded-All-MPR problem*. More precisely, let *k* be the maximum allowed host switch distance. Adding to the dynamic programming procedure a test for checking the distance of a host switch, we obtain an algorithm whose time complexity is $O(nm\dot {2^{k}})$. However, for a constant value of *k*, this complexity remains in *O*(*nm*). Observe that if we do not require the *k* bound on the distance of the switches, one could easily replace the algorithm by the theoretically faster method of Bansal *et al.*

Finding one optimal solution requires keeping trace of a path in the matrix leading to the minimum cost. This is easily done by keeping in each *D*(*p*,*h*) cell not only the cost associated to it but a pair of pointers to one mapping for the children *p*_1_,*p*_2_ of *p* having led to such cost.

#### Enumerating all optimal solutions

**Figure 2:**
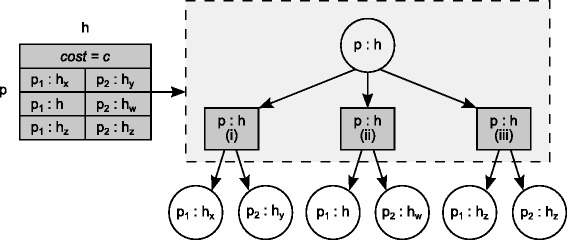
**Local tree structure for a given cell of** ***D*** **.** Schematic representation of the content of a cell in the dynamic programming matrix. Suppose the cell is related to the association *p*:*h* and let *p*_1_,*p*_2_ be the two children of *p*. One single cell-root node is created to represent the association *p*:*h* (the circular node in the picture). This association has a local minimum cost *c* that can be obtained in different ways, that is choosing different associations for *p*1 and *p*2. Each equivalent alternative is represented by a node (squared in the picture). The number of alternatives is variable. In this example, we have three alternatives: (i) *p*_1_ is mapped into *h*_*x*_ and *p*_2_ is mapped into *h*_*y*_; (ii) *p*_1_ is mapped into *h* and *p*_2_ is mapped into *h*_*w*_; and, (iii) *p*_1_ and *p*_2_ are both mapped into *h*_*z*_. Each one of these alternatives, combined with the mapping of *p* into *h* give the same local minimum cost *c*. Notice that, *h*, *h*_*w*_, *h*_*x*_, *h*_*y*_, and *h*_*z*_ are distinct nodes of the host tree.

**Figure 3:**
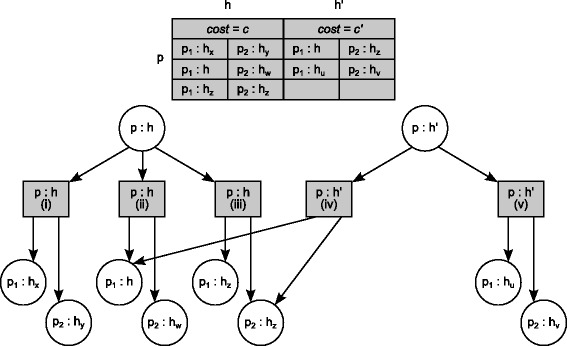
**Multiple sub-solutions.** The tree structure allows us to save the information in an efficient way. Each sub-solution corresponds to a subtree and there is no need to duplicate it each time it appears in a solution. In particular, only one node is created for each association and if two different alternatives share this association, the respective (square) nodes will point exactly at the same (circular) node. In this example, the mapping of *p* into *h* has the same alternatives (i), (ii) and (iii) as depicted in Figure 2. The association of *p* with *h* ^′^ has local minimum cost of *c* ^′^ and can be obtained by two mappings of *p*_1_ and *p*_2_: (iv) *p*_1_ is mapped into *h* and *p*_2_ is mapped into *h*_*z*_; and (v) *p*_1_ is mapped into *h*_*u*_ and *p*_2_ is mapped into *h*_*v*_. Notice that, *h*, *h* ^′^, *h*_*u*_, *h*_*v*_, *h*_*w*_, *h*_*x*_, *h*_*y*_, and *h*_*z*_ are distinct nodes of the host tree.

To enumerate all solutions, we need to keep more information. This can be done using *O*(*n*^3^*m*) space instead of *O*(*nm*). Consider a cell *c*=*D*(*p*,*h*) of the dynamic programming matrix *D*. Besides the numerical value corresponding to the cost of an optimal solution obtained by mapping *p* to *h*, the cell now also contains a list of pairs of pointers, one to each of the mappings of the children *p*_1_ and *p*_2_ of *p* having led to the cost of an optimal sub-solution that mapped *p* to *h*. Clearly the size of such a list is *O*(*n*^2^) in the worst case. The set of all pointers for *D* naturally form a DAG-like structure that is driven by the topology of the parasite tree. Figure 2 shows the information contained in cell *c*=*D*(*p*,*h*) of the matrix (left side). This may be also visualised in the form of a local tree (right side of the same figure) with the parent vertex *c* as the root which corresponds to the mapping of *p* to *h* (denoted in the figure by *p*:*h*) and one child for each alternative solution leading to that mapping (rectangle vertices in the figure). Each such alternative solution in turn corresponds to a pair of pointers, to two circle vertices which represent, in each case, a different pair of mappings of the children *p*_1_ and *p*_2_ of *p* which is equivalent in terms of cost (and is optimal). The circle vertices thus correspond to other cells of the matrix *D* which contain a similar local tree. Notice that more than one sub-solution may refer to a same mapping as indicated in Figure 3, thus leading to a DAG structure when the set of all solutions is considered and representing a compact structure for containing them all. Once built during the first pass over *D*, this DAG is then visited in pre-order to iteratively extract each such solution in turn. For more details concerning both algorithms, see Additional file 1.

#### Complexity analysis

The space complexity of EUCALYPT is *O*(*n*^3^*m*). For each of the *mn* steps of the dynamic programming procedure, we create at most *n*^2^ objects. All the additional structures used to iterate over this matrix have size *O*(*n*). To evaluate the time complexity of the whole enumeration process, we separate the time needed for filling matrix *D*, and the time for traversing it in order to produce a single solution, or to enumerate all of them. The number of steps needed for filling the matrix the first time is *O*(*n*^3^*m*) because each cell may contain, in the worst case, a list of *n*^2^ pairs of pointers. Since the height of the DAG is bounded by 2*m*, it can be traversed using only a linear size support structure. Moreover, at each iteration that leads to one solution, a subtree of size 2*m* of the DAG is visited. In particular, each time we are visiting a parent vertex, we need to add its mapping to the current solution and then follow the DAG looking for the mappings of its two children. An entire solution (which is composed by *m* mappings) is complete when at most 2 vertices (one parent and one sub-solution) have been visited for each vertex of *P*. This guarantees that once we produce the first solution, obtaining each one of the others in turn requires only linear time and linear space.

Finally, it is possible to enumerate only time-feasible solutions. To this purpose we have implemented a time-feasibility test defined in [14] which has a time complexity of *O*(*n*^2^).

## Results and discussion

### Datasets

To test EUCALYPT, we selected 12 datasets from the literature. As we are mostly interested in host-parasite systems, the first 10 datasets concern such relations: EC - Encyrtidae & Coccidae [23], GL - Gopher & Lice [24], SC - Seabirds & Chewing Lice [25], RP - Rodents & Pinworms [26], SCF - Smut Fungi & Caryophillaceus plants [27], PLML - Pelican Lice ML [28] (the trees are generated through a maximum likelihood approach), PLMP - Pelican Lice MP [28] (the trees are generated through a maximum parsimony approach), RH - Rodents & Hantaviruses [29], PP - Primates & Pinworm [30], and FD - Fishs and Dactylogyrus [31].

In addition, we used a dataset of our own which corresponds to arthropod hosts and a bacterium genus, *Wolbachia*, living inside the cells of their hosts [32,33]. The datasets were chosen to provide a variety in terms of size of the host and parasite trees: those from the literature are relatively small (from 7 to 100 leaves), while our own data provide an example of much bigger host and parasite trees, each having 387 leaves. Moreover, we were careful that the selected datasets cover, as much as possible, a range of situations in terms of coevolution and of the expected frequencies of each event. Finally, since EUCALYPT can be applied to any type of datasets compatible with the model, we also tested it on a genes-species dataset from [17] that had previously been used by [20]. The dataset has 3983 unrooted gene trees.

To be able to run our algorithm on this dataset, we rooted the trees using an approach similar to [20]: for each possible rooted version of the unrooted gene tree, we consider the optimal cost of the reconciliation and choose the rooting that has minimum cost among all. In this paper, we only show the results concerning 4 datasets: COG2085, COG3715, COG4964, COG4965. The choice of these datasets is motivated by the fact that they show different behaviour, in particular as concerns the *k-bounded-All-MPR* problem.

The datasets from the literature are given in Table 1, together with the number of leaves in the host and parasite trees.

**Table 1:** **Number of solutions found by each one of the programs** CORE-PA, NOTUNG and EUCALYPT

**Dataset**	**Leaves**		**Cost vector**	**Reconciliations**						
	**H**	**P**		**CORE-PA**			**EUCALYPT**			
				**#T**	**#C**	**#A**	**#T**	**#C**	**#A**	**#CA**
EC	7	10	〈0,1,1,1〉	16	6	10	16	6	10	5
			〈0,1,2,1〉	**14**	0	**14**	18	0	18	6
			〈0,2,3,1〉	**12**	0	**12**	16	0	16	4
GL	8	10	〈0,1,1,1〉	2	0	2	2	0	2	1
			〈0,1,2,1〉	2	0	2	2	0	2	1
			〈0,2,3,1〉	2	0	2	2	0	2	1
SC	11	14	〈0,1,1,1〉	1	0	1	1	0	1	1
			〈0,1,2,1〉	1	0	1	1	0	1	1
			〈0,2,3,1〉	1	0	1	1	0	1	1
RP	13	13	〈0,1,1,1〉	18	2	16	18	2	16	3
			〈0,1,2,1〉	3	1	2	3	1	2	1
			〈0,2,3,1〉	3	1	2	3	1	2	1
SFC	15	16	〈0,1,1,1〉	184	40	144	184	40	144	1
			〈0,1,2,1〉	40	40	0	40	40	0	0
			〈0,2,3,1〉	40	40	0	40	40	0	0
PLML	18	18	〈0,1,1,1〉	**158**	0	**158**	180	0	180	4
			〈0,1,2,1〉	2	0	2	2	0	2	1
			〈0,2,3,1〉	11	0	11	11	0	11	2
PLMP	18	18	〈0,1,1,1〉	2	0	2	2	0	2	1
			〈0,1,2,1〉	2	0	2	2	0	2	1
			〈0,2,3,1〉	**17**	0	**17**	18	0	18	2
RH	34	42	〈0,1,1,1〉	**32**	0	**32**	42	0	42	4
			〈0,1,2,1〉	**158**	**158**	0	2208	2208	0	0
			〈0,2,3,1〉	**22**	**22**	0	288	288	0	0
PP	36	41	〈0,1,1,1〉	1000*	0	**1000**	5120	0	5120	4
			〈0,1,2,1〉	**11**	0	**11**	72	0	72	2
			〈0,2,3,1〉	**11**	0	**11**	72	0	72	2
FD	20	51	〈0,1,1,1〉	1000*	**282**	**718**	25184	1792	23392	11
			〈0,1,2,1〉	**108**	**44**	**64**	408	132	276	5
			〈0,2,3,1〉	**22**	**22**	0	80	80	0	0
COG2085	100	44	〈0,1,1,1〉	1000*	**0**	**1000**	44544	2304	42240	3
			〈0,1,2,1〉	1000*	**0**	**1000**	37568	480	37088	7
			〈0,2,3,1〉	**888**	0	**888**	46656	0	46656	4
COG3715	100	40	〈0,1,1,1〉	1000*	**1000**	**0**	1172598	1155958	16640	6
			〈0,1,2,1〉	9	9	0	9	9	0	0
			〈0,2,3,1〉	**13**	**13**	0	33	33	0	0
COG4964	100	27	〈0,1,1,1〉	**85**	**85**	0	224	224	0	0
			〈0,1,2,1〉	**13**	**13**	0	36	36	0	0
			〈0,2,3,1〉	**17**	**17**	0	54	54	0	0
COG4965	100	30	〈0,1,1,1〉	1000*	**408**	**592**	17408	5632	11776	2
			〈0,1,2,1〉	**141**	0	**141**	640	0	640	2
			〈0,2,3,1〉	1000*	**276**	**724**	6528	1408	5120	2

Number of solutions found by each one of the programs CORE-PA, NOTUNG and EUCALYPT for each dataset and each cost vector 〈*c*_*c*_,*c*_*d*_,*c*_*s*_,*c*_*l*_〉. For EUCALYPT and CORE-PA the columns represent: #T = total number of optimal solutions, #C = total number of cyclic solutions and #A = total number of acyclic solutions. In all cases #A is always equal for both NOTUNG and EUCALYPT. For EUCALYPT the column #CA denotes the number of event classes in the set of acyclic solutions. CORE-PA limits to 1000 the total number of enumerated solutions and these cases are denoted by the symbol ∗. Bold numbers indicate the cases where the number of solutions produced by CORE-PA differs from the one found by EUCALYPT.

### Comparison with CORE-PA and NOTUNG

In Table 1, we compare EUCALYPT to CORE-PA and NOTUNG for some cost vectors that commonly appear in the literature.

As mentioned in the introduction, CORE-PA does not always enumerate all solutions, as shown in Table 1. In the Additional file 1, we give an explicit example from one of the datasets used (“Smut Fungi & Caryophillaceus plants” [27] with cost vector 〈0,1,1,1〉) where indeed EUCALYPT finds more (correct) solutions, some of which are acyclic, that are time-feasible. The same result is observed for other datasets (examples not shown).

Notice that sometimes when enumerating optimal reconciliations, CORE-PA outputs the same one more than once. In all our tests, we were never able to obtain more than 1000 different reconciliations with CORE-PA. This explains the presence of the value 1000 twice in Table 1 and Table 2 (each time indicated by a ^∗^) while EUCALYPT for the same datasets and cost vectors finds many more solutions.

**Table 2:** **Number of solutions found by the programs** CORE-PA and EUCALYPT

**Dataset**	**Leaves**		**Cost vector**	**Reconciliations**						
	**H**	**P**		**CORE-PA**			**EUCALYPT**			
				**#T**	**#C**	**#A**	**#T**	**#C**	**#A**	**#CA**
EC	7	10	〈−1,1,1,1〉	2	0	2	2	0	2	1
			〈0,1,1,0〉	**18**	0	**18**	24	0	24	8
GL	8	10	〈−1,1,1,1〉	2	0	2	2	0	2	1
			〈0,1,1,0〉	12	0	12	12	0	12	5
SC	11	14	〈−1,1,1,1〉	1	0	0	1	0	1	1
			〈0,1,1,0〉	**82**	2	**80**	113	3	110	18
RP	13	13	〈−1,1,1,1〉	3	1	2	3	1	2	1
			〈0,1,1,0〉	**69**	**25**	**44**	117	45	72	29
SFC	15	16	〈−1,1,1,1〉	40	40	0	40	40	0	0
			〈0,1,1,0〉	1000*	**741**	**259**	6332	5069	1263	81
PLML	18	18	〈−1,1,1,1〉	2	0	0	2	0	2	1
			〈0,1,1,0〉	**45**	**2**	**43**	448	28	420	16
PLMP	18	18	〈−1,1,1,1〉	2	0	0	2	0	2	1
			〈0,1,1,0〉	**147**	0	**147**	262	0	262	34
RH	34	42	〈−1,1,1,1〉	**197**	**197**	0	1056	1056	0	0
			〈0,1,1,0〉	1000*	**0**	**1000**	4080384	310284	3770100	275
PP	36	41	〈−1,1,1,1〉	**17**	0	**17**	144	0	144	2
			〈0,1,1,0〉	**182**	**8**	**174**	498960	55440	443520	129
FD	20	51	〈−1,1,1,1〉	**196**	**86**	**110**	944	368	576	7
			〈0,1,1,0〉	1000*	**1000**	**0**	1.5×10^15^	*	*	*
COG2085	100	44	〈−1,1,1,1〉	1000*	**0**	**1000**	109056	26496	82560	3
			〈0,1,1,0〉	1000*	**0**	**1000**	3.5×10^11^	*	*	*
COG3715	100	40	〈−1,1,1,1〉	**869**	**869**	0	63360	63360	0	0
			〈0,1,1,0〉	1000*	**0**	**1000**	1.2×10^12^	*	*	*
COG4964	100	27	〈−1,1,1,1〉	**13**	**13**	0	36	36	0	0
			〈0,1,1,0〉	1000*	**0**	**1000**	8586842	2603598	5983244	300
COG4965	100	30	〈−1,1,1,1〉	1000*	**335**	**665**	44800	13312	31488	5
			〈0,1,1,0〉	1000*	**0**	**1000**	907176	387192	519984	208

Number of solutions found by the programs CORE-PA and EUCALYPT for each dataset and each cost vector 〈*c*_*c*_,*c*_*d*_,*c*_*s*_,*c*_*l*_〉. The columns represent: #T = total number of optimal solutions, #C = total number of cyclic solutions and #A = total number of acyclic solutions. For EUCALYPT the column #CA denotes the number of event classes in the set of acyclic solutions. CORE-PA limits to 1000 the total number of enumerated solutions and these cases are denoted by the symbol ∗. Bold numbers indicate the cases where the number of solutions produced by CORE-PA differs from the one found by EUCALYPT.

NOTUNG generates only time-feasible reconciliations and their number coincides with the result of EUCALYPT for all the datasets used. However, NOTUNG imposes some restrictions on the cost values. Indeed, the cost of a cospeciation is always assumed to be equal to 0 and the cost of a loss positive.

### Non positive cost vectors

No assumption needs to be made by EUCALYPT concerning the cost values: it thus allows negative ones while cospeciation and loss may have any arbitrary cost. As already mentioned, in this case we can compare it only with CORE-PA. The results of these experiments are presented in Table 2. In almost all of the cases, CORE-PA does correctly determine the total number of (un)feasible optimal solutions.

### Results of **EUCALYPT** and discussion

The results obtained by EUCALYPT and presented in Tables 1 and 2 are striking for various reasons.

First, we observe that when the size of the tree increases, in most cases the total number of optimal solutions also increases. However, this does not hold for the number of time-feasible optimal solutions. For instance, according to the results given in Table 1, for the dataset EC having 7 and 10 leaves, the number of time-feasible optimal solutions is much higher than for the case of dataset COG4964 (having 100 and 27 leaves). Even for the same dataset, this number can be reduced significantly depending on the cost vector. In particular when the cost of the losses is 0, the number of optimal solutions can be huge even for relatively small datasets, such as for example FD (20-51 leaves). This makes it practically impossible to check the time-feasibility of each of them (this explains the presence of a * in some cells of Tables 1 and 2). Thus, it seems that the cost vector and the topology of the trees together with the mapping of the leaves play a more important role in the total number of time-feasible solutions.

We also tested EUCALYPT on the much bigger trees of *Wolbachia* and the arthropods. Due to limitations in space and time, we could not enumerate all optimal solutions because their number is huge: 1.01×10^47^ for cost vector 〈−1,1,1,1〉, 3.87×10^136^ for cost vector 〈0,1,1,0〉, 3.19×10^48^ for cost vector 〈0,1,1,1〉, and finally 1.01×10^47^ for cost vector 〈0,1,2,1〉. We did however enumerate optimal solutions until one was produced that was found acyclic.

For the cost vector 〈0,1,1,1〉, the first produced optimal solution was already acyclic as were those that were enumerated next, hinting to the possibility that the proportion of acyclic solutions is high among all optimal ones. For the remaining cost vectors, the initial optimal solutions enumerated by EUCALYPT were indeed all cyclic, and given their number and the time required by each acyclicity test, we stopped the process of checking after one week. Two cases are then possible: either there are no acyclic solutions meaning optimal ones have a higher cost; or the proportion of acyclic solutions is low among all optimal ones.

The results confirm once again that the number of optimal reconciliations can be huge. Moreover, the problem remains even if we restrict the results to only time-feasible solutions. In order to deal with this huge set of solutions, we propose to group them in classes depending on the number of events observed. As shown in Tables 1 and 2, the number of classes in a set of time-feasible optimal solutions is significantly smaller compared to the size of the set itself.

For instance, for dataset EC and vector 〈0,1,1,1〉, the 10 optimal time-feasible solutions are split in 5 classes 〈*#*_*c*_,*#*_*d*_,*#*_*s*_,*#*_*l*_〉 as follows: 1 for 〈4,1,4,1〉, 3 for 〈4,0,5,1〉, 2 for 〈5,0,4,2〉, 2 for 〈3,1,5,0〉 and, 2 for 〈3,0,6,0〉. For dataset RP and vector 〈0,1,1,1〉, the 16 optimal time-feasible solutions are split in 3 classes: 4 for 〈7,0,5,3〉, 2 for 〈4,0,5,1〉 and, 10 for 〈6,0,6,2〉. Even more interestingly, for dataset SFC and vector 〈0,1,1,1〉, the 144 optimal time-feasible solutions belong to a unique class: 〈4,0,11,0〉. For more details on this type of analysis, we refer to Additional file 1.

Finally, we want to call attention to the fact that, in many cases of datasets and cost vectors, there is no optimal solution that is time-feasible. Indeed, in the case of the datasets from [17], from 3983 trees we choose 429 with between 20 and 50 leaves for which the total number of optimal reconciliations is less than 10000. Among these 429 trees (rooted according to the one that leads to a minimum total cost of the reconciliation) and using the vector 〈0,2,3,1〉, 233 (i.e. more than half) have no time-feasible solutions. To deal with this problem, we consider the restriction when the host switches are constrained to have bounded distance.

### k-bounded-All-MPR problem

The main concern of this section is to discuss the effect of bounding the distance of the host switch events. The variables that will be defined here must be considered relative to a fixed dataset and a fixed cost vector. We denote by *S*(*k*) the set of optimal solutions obtained when the maximum distance allowed for a host switch is *k*, and denote by *opt*_*k*_ their cost. We also denote by *opt*^∗^ the optimum cost of an acyclic reconciliation (without any bound on the host switch distance): observe that this value is in general NP-hard to determine. Clearly, if *S*(*∞*) contains at least one time-feasible solution then *opt*_*∞*_=*opt*^∗^. However, this is not always the case (see Table 1 for some examples): let us then consider the case where *opt*_*∞*_<*opt*^∗^. We are now interested in finding an upper bound on the value of *opt*^∗^ by making use of the possibility given by EUCALYPT to limit the distance of switches. To this purpose, we define *k*_*A*_ as the biggest value of *k* for which *S*(*k*) contains at least one acyclic solution (in general, $opt_{\infty } < {opt}^{*} \leq {opt}_{k_{A}}$): observe that in the case of integer cost values, when $opt_{\infty }-opt_{k_{A}}=1$, we have that $opt_{k_{A}}$ coincides with *opt*^∗^. We can determine *k*_*A*_ as follows: for every optimal reconciliation in *S*(*∞*), we keep track of the longest distance observed for a switch and denote by *k*_*start*_ the smallest value observed among all optimal solutions; then starting from *k*_*start*_, we decrement this value until at least one time-feasible solution is found. It is interesting to note that *k*_*A*_ is always close to the starting value *k*_*start*_, and that it frequently happens that $opt_{\infty }-opt_{k_{A}}=1$. This shows that the method is efficient in practice as we do not have to check too many values before finding time-feasible optimal solutions. In particular, we applied this idea to some cases where no time-feasible solutions were found: the results are shown in Table 3.

**Table 3:** **Searching for time-feasible solutions by varying** ***k***

**Dataset**	**Costvector**								
	**〈−1,1,1,1〉**			**〈0,1,2,1〉**			**〈0,2,3,1〉**		
	***k***_***start***_ ***→k***_***A***_	$\boldsymbol {o \rightarrow o_{k_{A}}}$	***#A***	***k***_***start***_ ***→k***_***A***_	$\boldsymbol {o \rightarrow o_{k_{A}}}$	***#A***	***k***_***start***_ ***→k***_***A***_	$\boldsymbol {o \rightarrow o_{k_{A}}}$	***#A***
SFC	7→6	6→7	16	7→6	21→22	16	7→5	31→35	12
RH	6→5	8→12	16	6→5	43→48	192	6→5	62→68	48
COG3715	13→12	10→11	288	22→6	51→176	6	22→6	80→206	2
COG4964	22→4	20→208	30	13→12	33→34	288	13→12	49→50	288

For some datasets (SFC, RH, COG3715 and, COG4964), the number of optimal time-feasible solutions is zero when reconciliations are obtained by using a given cost vector and unbounded *k*. After identifying *k*_*start*_ (minimum *k* whose optimal cost *o* is equal to the optimal cost obtained for unbounded *k*), we decremented *k* until *k*_*A*_ (maximum *k* which generates acyclic solutions) is found. For each pair (dataset, cost vector), the following values are given: the decrement of the bound (from *k*_*start*_ to *k*_*A*_), the new optimum found (from *o* to *o*_*A*_) and the new number of acyclic solutions (*#* *A*).

Even in the case where *S*(*∞*) contains already some time-feasible solutions, bounding the distance of the switches remains interesting because the number of such solutions can be too large to handle. The basic idea is to choose a value *k*^′^, with *∞*>*k*^′^≥*k*_*start*_, and to focus our attention only on *S*(*k*^′^): in this way, the optimal cost of the solutions is preserved and |*S*(*k*^′^)|≤|*S*(*∞*)|. In real situations, choosing *k*^′^ must be driven by some *ad hoc* biological consideration: however, in our case, we decided to fix *k*^′^ to the value that is nearest to *k*_*start*_ for which the optimal cost does not change and the number of time-feasible solutions is strictly positive. Some results are shown in Table 4: it is worth observing that *k*^′^ always either coincides or is only a few steps away from *k*_*start*_, which again shows the efficiency of the method in practice.

**Table 4:** **Reducing the number of optimal time-feasible solutions by bounding** ***k***

**Dataset**	**Costvector**								
	**〈−1,1,1,1〉**			**〈0,1,2,1〉**			**〈0,2,3,1〉**		
	***k*** **/** ***k*** ^***′***^	***#*** ***A*** ***C*** **/** ***#*** ***A*** ***C*** ^***′***^	***#*** ***T*** **/** ***#*** ***T*** ^***′***^	***k*** **/** ***k*** ^***′***^	***#*** ***A*** ***C*** **/** ***#*** ***A*** ***C*** ^***′***^	***#*** ***T*** **/** ***#*** ***T*** ^***′***^	***k*** **/** ***k*** ^***′***^	***#*** ***A*** ***C*** **/** ***#*** ***A*** ***C*** ^***′***^	***#*** ***T*** **/** ***#*** ***T*** ^***′***^
EC	3/3	2/2	2/2	3/3	18/16	18/16	3/3	16/16	16/16
GL	4/4	2/2	2/2	4/4	2/2	2/2	4/4	2/2	2/2
SC	6/6	1/1	1/1	6/6	1/1	1/1	6/6	1/1	1/1
RP	9/9	2/2	3/3	8/8	2/2	8/8	2/2	2/2	3/3
PMP	6/6	2/2	2/2	6/6	2/2	2/2	5/5	11/4	11/4
PML	5/5	2/2	2/2	5/5	2/2	2/2	3/3	18/6	18/6
PP	4/4	144/96	144/96	4/4	72/48	72/48	4/4	72/48	72/48
FD	9/10	576/240	944/512	9/9	276/4	408/8	−	−	−
COG2085	14/14	82560/9408	109056/9408	14/14	37088/4032	37568/4032	14/14	46656/5184	46656/5184
COG4965	16/16	31448/15744	44800/22400	16/16	640/320	640/320	13/16	5120/2560	6528/3328

For some datasets, the number of optimal time-feasible solutions may be huge when *k* is unbounded. In some cases, however, by introducing a bound on *k* we can greatly reduce the number of time-feasible solutions while keeping their optimality. For all datasets whose number of acyclic solutions is positive for unbounded *k*, we identified *k*_*start*_ (minimum *k* whose optimal cost is equal to the optimal cost obtained for unbounded *k*) and we searched for the minimum *k* ^′^≥*k*_*start*_ whose number of acyclic solutions is non zero. We executed this procedure for every pair (dataset, cost vector) for which the number of optimal acyclic solutions is positive. In the first column, the values for *k*=*k*_*start*_ and *k* ^′^ are given. *#* *A* *C*/*#* *A* *C* ^′^ denotes the number of optimal acyclic solutions for the case when the switches are unbounded and the case when they are bounded by *k* ^′^, respectively. The same relation is shown for the total number of optimal solutions in the column *#* *T*/*#* *T* ^′^.

## Conclusions

We presented in this paper a software, EUCALYPT, that can find one optimal reconciliation of a pair of host and parasite trees, can compute the number of all optimal solutions, and can enumerate them all. The first two problems are handled in polynomial time, while the enumeration has a polynomial delay complexity. EUCALYPT also displays the classes of solutions observed, where two solutions are in a same class if the number of each event in the two is the same. We show that although the number of classes of solutions may be considerably smaller than the total number of optimal reconciliations, it nevertheless may remain very high even for relatively small trees. Finally, we introduced the *k-bounded-All-MPR* problem and showed how it could be applied either to find optimal time-feasible solutions when the parsimonious method found none, or to reduce their number if this is too large to be handled in practice for further analysis. EUCALYPT takes a nexus file as input and generates all the information related to the reconciliations described in the paper. The datasets used in this paper are also available on the website of the software.

All the results of this study point to the necessity of introducing new criteria besides parsimony in the model for an optimal reconciliation. The idea of imposing an evolutionary distance to the host switches is one possible criterion when it can be justified from a biological point of view. Other types of information, such as for instance geographic, might enable also to indicate that certain mappings of internal vertices are impossible. One next improvement of EUCALYPT will therefore be to allow the user to indicate that certain associations of internal vertices should be forbidden. Finally, it remains an open question whether some such additional criteria could change the complexity of the reconciliation problem when only feasible (acyclic) solutions are to be found or enumerated.

## Availability and requirements

- **Project name:** Eucalypt
- **Project home page:**http://eucalypt.gforge.inria.fr/
- **Operating system(s):** Any
- **Programming languages:** Java 1.6
- **Other requirements:** None
- **License:** CeCILL
- **Any restrictions to use by non-academics:** None.

## Additional file

Additional file 1
**Model, algorithms and experimental results.** Description of the model and pseudo-code for the algorithms for finding one optimal solution (Algorithm 1) and enumerating all solutions (Algorithm 2). Collection of plots showing the characteristics of the set of optimal solutions for the studied datasets.
