# Supplementary material for: EUCALYPT: efficient tree reconciliation enumerator
Source: Algorithms Mol Biol. 2015 Jan 23;10:3. doi: 10.1186/s13015-014-0031-3 (PMC4310143; doi:10.1186/s13015-014-0031-3)
Supplement: Additional file 1 — Model, algorithms and experimental results. Description of the model and pseudo-code for the algorithms for finding one optimal solution (Algorithm 1) and enumerating all solutions (Algorithm 2). Collection of plots showing the characteristics of the set of optimal solutions for the studied datasets. [file 13015_2014_31_MOESM1_ESM.pdf]

EUCALYPT: Efficient tree reconciliation enumerator

Beatrice Donati, Christian Baudet*, Blerina Sinaimeri,
Pierluigi Crescenzi, and Marie-France Sagot*

1 Supplementary Material

We provide below the details of the DTL-model and the pseudo-code for the two algorithms presented in the paper.

The model

Henceforth, by a phylogenetic tree T, we thus mean a rooted tree with labelled leaves and where the root has in-degree 0 and out-degree 2, the leaves have out-degree 0 and in-degree 1 and every other vertex has in-degree 1 and out-degree 2. For such a tree T, the set of vertices is denoted by $V(T)$, the set of arcs by $A(T)$, and the set of leaves by $L(T)$. The root of T is denoted by $r(T)$. Given an arc $a = (v, w) \in A(T)$, going from v to w, we call its *head*, denoted by $h(a)$, the vertex w and its *tail*, denoted by $t(a)$, the vertex v. For a vertex $v \in V(T)$, we define the set of *descendants* of v, denoted by $Des(v)$, as the set of vertices in the subtree of T rooted at v. Similarly, the set of *ancestors* of v, denoted by $Anc(v)$, is the set of vertices in the unique path from the root of T to v, including the root and v. For a vertex $v \in V(T)$ different from the root, we call its *parent*, denoted by $par(v)$, the vertex x for which there is the arc $(x, v) \in A(T)$. We denote by $\texttt{lca}(v, w)$ the least common ancestor of v, w in T. Finally, we denote by $\geq$ the partial order induced by the ancerstorship relation in the tree. Formally, for $x, y \in V(T)$, we say that $x \geq y$ if $x \in Anc(y)$. If neither $x \in Anc(y)$ nor $y \in Anc(x)$, the vertices are said to be *incomparable*.

The model of host-parasite evolution we rely on in this paper is the event-based model presented by Tofigh *et al.* [1], and later further analysed by Bansal *et al.* [2]. Let H, P be the phylogenetic trees for the host and parasite species respectively. We define ϕ as a function from the leaves of P to the leaves of H that represents the association between currently living host species and parasites. Such association is an input of our algorithm, together with the trees themselves. In our model, we allow each parasite to be related to one and only one host, while a host can be related to zero, one, or more than one parasite. More formally, ϕ is thus a function which may be neither surjective nor injective.

In studying the co-evolution of hosts and parasites, the following set of events are generally allowed to take place: (a) co-speciation: the host and parasite speciate concurrently, (b) duplication: the parasite speciates independently from the host and both new species of parasites remain with the host, (c) loss: the host speciates but the parasite does not, and (d) host-switch: the parasite speciates but one of the new parasite species switches (jumps) to another host.

*Corresponding authors: christian.baudet@inria.fr, marie-france.sagot@inria.fr

A *reconciliation* γ is a function $\gamma : V(P) \to V(H)$ that is an extension of ϕ. In particular γ partitions the set $V(P)$ into three sets Σ, Δ, and Θ, and a subset Ξ of $A(P)$, for which the following hold [1]:

1. For any $p \in L(P)$, $\gamma(p) = \phi(p)$ (γ extends ϕ).
2. For any internal vertex $p \in V(P) - L(P)$ with children p_1 and p_2:
   (a) $\texttt{lca}(\gamma(p), \gamma(p_i)) \geq \gamma(p_i)$, for $i = 1, 2$ (a child cannot be mapped in an ancestor of the father).
   (b) $\texttt{lca}(\gamma(p), \gamma(p_1)) = \gamma(p)$ or $\texttt{lca}(\gamma(p), \gamma(p_2))) = \gamma(p)$ (one of the two children is mapped in the subtree rooted at the father).
3. For any $(p_1, p_2) \in \Xi \Leftrightarrow \texttt{lca}(\gamma(p_1), \gamma(p_2)) \notin \{\gamma(p_1), \gamma(p_2)\}$ (the arc (p_1, p_2) is an arc denoting a switch event).
4. For any $p \in V(P) - L(P)$ with children p_1 and p_2:
   (a) $p \in \Theta \Leftrightarrow (p, p_1) \in \Xi$ or $(p, p_2) \in \Xi$ (p is associated to a switch event.
   (b) $p \in \Delta \Leftrightarrow \texttt{lca}(\gamma(p_1), \gamma(p_2)) \in \{\gamma(p_1), \gamma(p_2)\}$ (the children are mapped to comparable vertices and p is associated to a duplication event).
   (c) $p \in \Sigma \Leftrightarrow \texttt{lca}(\gamma(p_1), \gamma(p_2)) = \gamma(p)$ and $\gamma(p_1)$ and $\gamma(p_2)$ are incomparable and p is associated to a co-speciation event.

The sets Σ, Δ, and Θ correspond to the vertices of P associated to, respectively, co-speciations, duplications, and host-switches, while the set Ξ corresponds to the arcs associated to host-switches. Finally, losses are identified by a multi-set $\Lambda \subseteq V(H)$ containing all the vertices $h \in V(H)$ that are in the path from the image of a vertex in $V(P)$ and the image of one of its children.

The triple $S = \langle H, P, \gamma \rangle$ is said to be a *scenario* or simply a reconciliation. Given a *vector* $\langle c_c, c_d, c_s, c_l \rangle$ of non negative real values that correspond to the cost of each type of event, the *cost* of a reconciliation is equal to $c_c|\Sigma| + c_d|\Delta| + c_s|\Theta| + c_l|\Lambda|$.

Finally, host switches can introduce an incompatibility due to the temporal constraints imposed by the host and parasite trees, as well as by the reconciliation itself. Determining whether a reconciliation is time-feasible can be done in polynomial time [3]. It is common to refer to a time-feasible (unfeasible) solution as acyclic (cyclic).

Given a riconciliation $\gamma : V(P) \to V(H)$ we build the digraph $G = (V_G, E_G)$ where: $V_G = V_H$ and the set of edges E_G is defined as follows:

- $E_G = E_H \cup$ for all the couples of transfer edges $(u, v)(u', v')$ for which $u \in Ancestors(u')$:
  - $(p(\gamma(u)), \gamma(u'))$
  - $(p(\gamma(u)), \gamma(v'))$
  - $(p(\gamma(v)), \gamma(u'))$
  - $(p(\gamma(v)), \gamma(v'))$

Note that when $(u, v) = (u', v')$ the edges to add are:

- $(p(\gamma(u)), \gamma(u))$ redundant
- $(p(\gamma(u)), \gamma(v'))$ (it creates a path from the ancestors of the donor to the recipient)

- $(p(\gamma(v)), \gamma(u'))$ (it creates a path from the ancestors of the recipient to the donor)
- $(p(\gamma(v)), \gamma(v))$ redundant

The reconciliation is time-feasible if the graph G does not contain any directed cycle. Intuitively the constraints are given by the following facts:

- From the ancestry induced by the parasite tree we know that u' happened strictly after u.
- The contemporary of $\gamma(u)$ and $\gamma(v)$ is induced by the transfer (u, v)
- Everything that has happened before $\gamma(u)$ has happened before $\gamma(v)$.

Finding one optimal solution

As for Bansal *et al.* [2], we make use of some auxiliary structures apart from the dynamic programming matrix D. We thus use another matrix of size m by n that will contain the optimal solutions of the subtrees and that we denote by D_{ST}. Formally, $D_{ST}(p, h)$ holds the cost of an optimal solution in which p is mapped to some vertex i in the host subtree rooted in h. We also use two vectors. One is denoted by $Switch_k$ gives, for each vertex h in H the set of vertices that are incomparable with h and are at a distance at most k from it. Formally, we have that $Switch_k(h) = \{g \in V(H) | d(h, g) \leq k \wedge lca(h, g) \notin \{h, g\}\}$ where $d(h, g)$ is the distance of vertex h to vertex g in H. The second vector, denoted by $cost_{switch}$, holds the cost of a switch to a given vertex h in H.

Enumerating all solutions

A pseudo-code for enumerating all solutions is given in Algorithm 2. We use an additional stack M in order to select which sub-solutions (local sub-trees) to add to the reconciliation that is currently being built. This stack is filled with couples of the form $\langle cell, index\rangle$). The function $M(cell)$ returns, in constant time, the couple $\langle cell, index\rangle$ at the top of M, if M is not empty.

Example of time-feasible optimal reconciliation not found by CoRe-Pa.

Here we provide a time-feasible reconciliation for the dataset "Smut Fungi & Caryophillaceus plants" [4] with cost vector $\angle 0, 1, 1, 1\rangle$, that is not found by CoRe-Pa.

Statistics on the optimal reconciliations

Here we show for each dataset and for each cost vector $\langle c_c, c_d, c_s, c_l\rangle$ some statistics concerning the optimal reconciliations.

Algorithm 1 Finding the cost of an optimal solution

Input: $< H, T, \phi >$ and a cost vector $\langle c_c, c_d, c_s, c_l \rangle$
Output: Optimal cost of the *k-bounded-All-MPR problem*

for $p \in V(P)$ and $h \in V(H)$ **do**
  Initialise $D(p,h), D_{ST}(p,h)$ to ∞
  Compute $Switch_k(h)$
end for
for $l \in L(P)$ **do**
  Initialise $D(l, \phi(l)) = 0$
  for $a \in Anc(\phi(l))$ **do**
    $D_{ST}(l,a) = c_l * d(a, \phi(l))$
  end for
end for
for $p \in V(P) - L(P)$ in post-order with children p_1, p_2 **do**
  for $h \in V(H)$ in post-order with children h_1, h_2 **do**
    if $h \in L(H)$ **then**
      $\delta_d \leftarrow c_d + c(p_1, h) + c(p_2, h)$
      for $g \in Switch_k(h)$ **do**
        $cost_{switch}(g) = c_s + min\{D(p_1, g) + D_{ST}(p_2, h), D(p_2, g) + D_{ST}(p_1, h)\}$
      end for
      $\delta_s \leftarrow min\{cost_{switch}(g) | g \in Switch_k(h)\}$
      $D(p,h) = min\{\delta_d, \delta_s\}$
      $D_{ST}(p,h) = D(p,h)$
    else
      $\delta_c \leftarrow min\{(c_c + D_{ST}(p_1, h_1) + D_{ST}(p_2, h_2)), (c_c + D_{ST}(p_1, h_2) + D_{ST}(p_2, h_1))\}$

$$\delta_d \leftarrow min \begin{cases} D(p_1, h) + D(p_2, h) \\ D(p_1, h) + D_{ST}(p_2, h_1) + c_l \\ D(p_1, h) + D_{ST}(p_2, h_2) + c_l \\ D(p_2, h) + D_{ST}(p_1, h_1) + c_l \\ D(p_2, h) + D_{ST}(p_1, h_2) + c_l \\ D_{ST}(p_1, h_1) + D_{ST}(p_2, h_1) + 2c_l \\ D_{ST}(p_1, h_2) + D_{ST}(p_2, h_2) + 2c_l \end{cases}$$

      for $g \in Switch_k(h)$ **do**
        $cost_{swicth}(g) = c_s + min\{D(p_1, g) + D_{ST}(p_2, h), D(p_2, g) + D_{ST}(p_1, h)\}$
      end for
      $\delta_s \leftarrow min\{cost_{switch}(g) | g \in Switch_k(h)\}$
      $D(p,h) = min\{\delta_c, \delta_d, \delta_s\}$.
      $D_{ST}(p,h) = min\{D(p,h), c_l + D_{ST}(p, h_1), c_l + D_{ST}(p, h_2)\}$
    end if
  end for
end for
return $min\{D(r(P), h) | h \in V(H)\}$

Algorithm 2 Enumerating all optimal solutions

Input: The dynamic programming matrix D
Output: All optimal solutions

for All cells *root* in D containing an optimal mapping of $r(P)$ (or the unique cell mapping $r(P)$ to $r(H)$) **do**
  $currentCell \leftarrow root$
  A stack $M \leftarrow [\emptyset]$ (to be filled with couples of the form $\langle cell, index\rangle$)
  do
    while $currentCell$!= null **do**
      if $|List(current)| \geq 1$ **then**
        //There are different sub-solutions for this mapping
        if $M(currentCell)$ is not in M **then**
          Push($\langle currentCell, 0\rangle$) in M
          $currentSubsolution \leftarrow 0^{th}$-element of $M(currentCell)$;
        else if $M(currentCell)$ is the last element of M **then**
          //In the final part of the solution I pass to consider the next option
          Pop($\langle currentCell, i\rangle$) from M
          Push($\langle currentCell, i+1\rangle$;) in M
          currentSubsolution $\leftarrow (i+1)th$-element of $M(currentCell)$
        else
          //In the first part of the current solution, the mappings are the same as for the previous one
          $\langle cell, index\rangle \leftarrow M(currentCell)$
          $currentSubsolution \leftarrow index^{th}$ element of $M(currentCell)$
        end if
      else
        //There is a unique possible sub-solution
        Add to the solution the mapping relative to $currentCell$
        $currentSubsolution \leftarrow 0th$-element of $List(currentCell)$
        //currentSubsolution is unique (or null if the vertex is a leaf.)
        $currentCell$ = the next vertex following the pointers of $currentSubsolution$ (in post-order)
      end if
      Output the solution
      Pop from M until the first couple $\langle s, i\rangle$ is found for which $i < |M(s)| - 1$ and the stack is not empty
    end while
  while M is not empty
end for

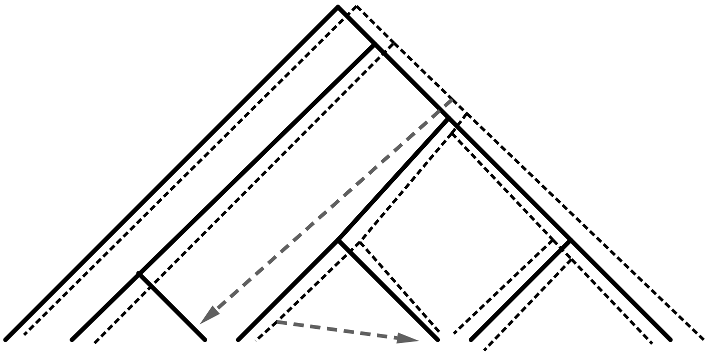

Figure 1: Example of a time-feasible reconciliation not found by CoRe-Pa

References

[1] Tofigh A, Hallett M, Lagergren J: **Simultaneous Identification of Duplications and Lateral Gene Transfers**. *IEEE/ACM Trans. on Comput. Biol. Bioinf.* 2011, **8**(2):517–535.

[2] Bansal MS, Alm E, Kellis M: **Efficient algorithms for the reconciliation problem with gene duplication, horizontal transfer and loss**. *Bioinformatics* 2012, **28**(12):i283–i291.

[3] Stolzer ML, Lai H, Xu M, Sathaye D, Vernot B, Durand D: **Inferring duplications, losses, transfers and incomplete lineage sorting with nonbinary species trees**. *Bioinformatics* 2012, **28**(18):i409–i415.

[4] Refrégier G, Gac M, Jabbour F, Widmer A, Shykoff J, Yockteng R, Hood M, Giraud T: **Cophylogeny of the anther smut fungi and their caryophyllaceous hosts: Prevalence of host shifts and importance of delimiting parasite species for inferring cospeciation**. *BMC Evol. Biol.* 2008, **8**:100.

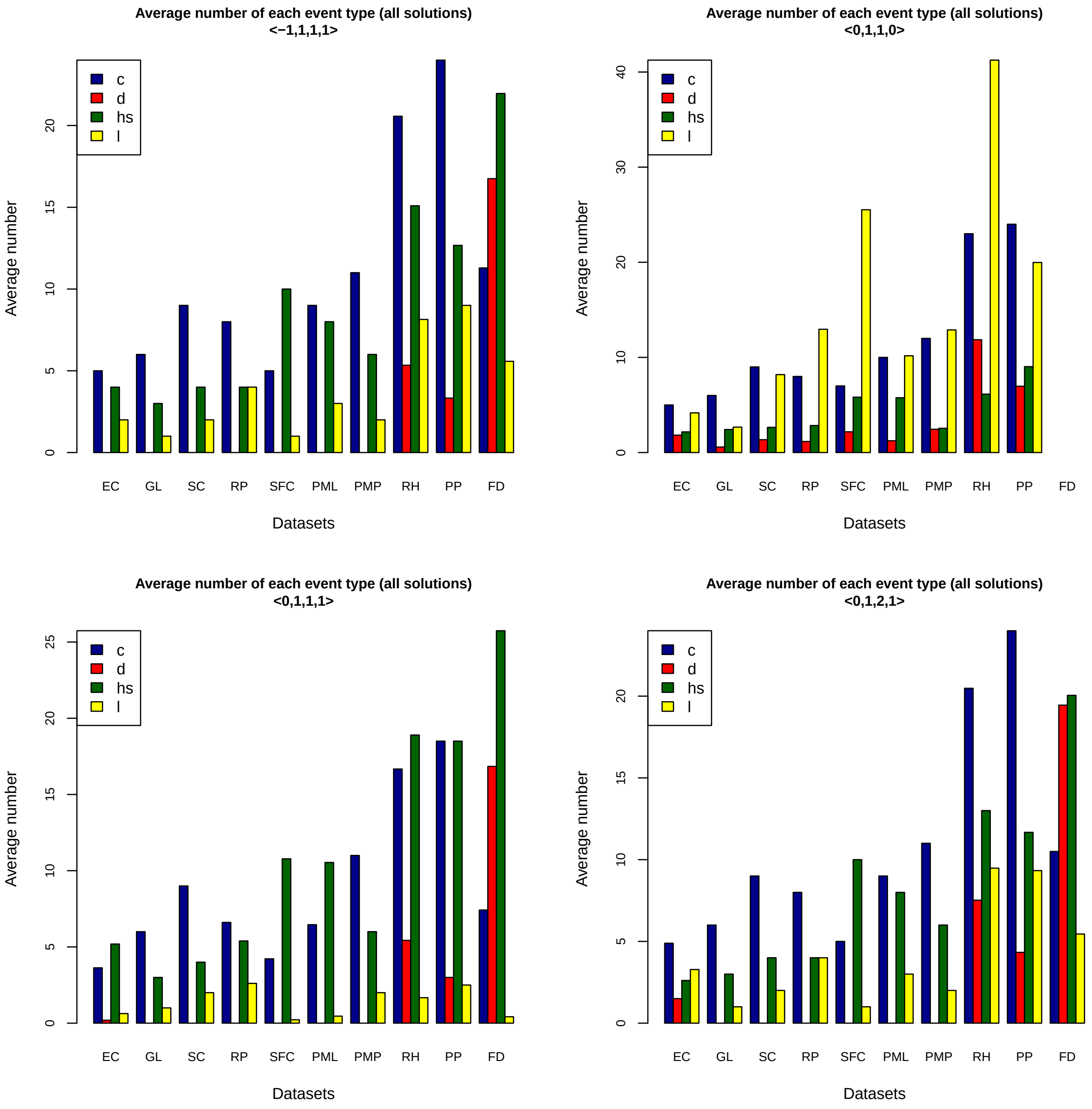

Figure 2: For each dataset we show the average number of each event in all optimal reconciliations.

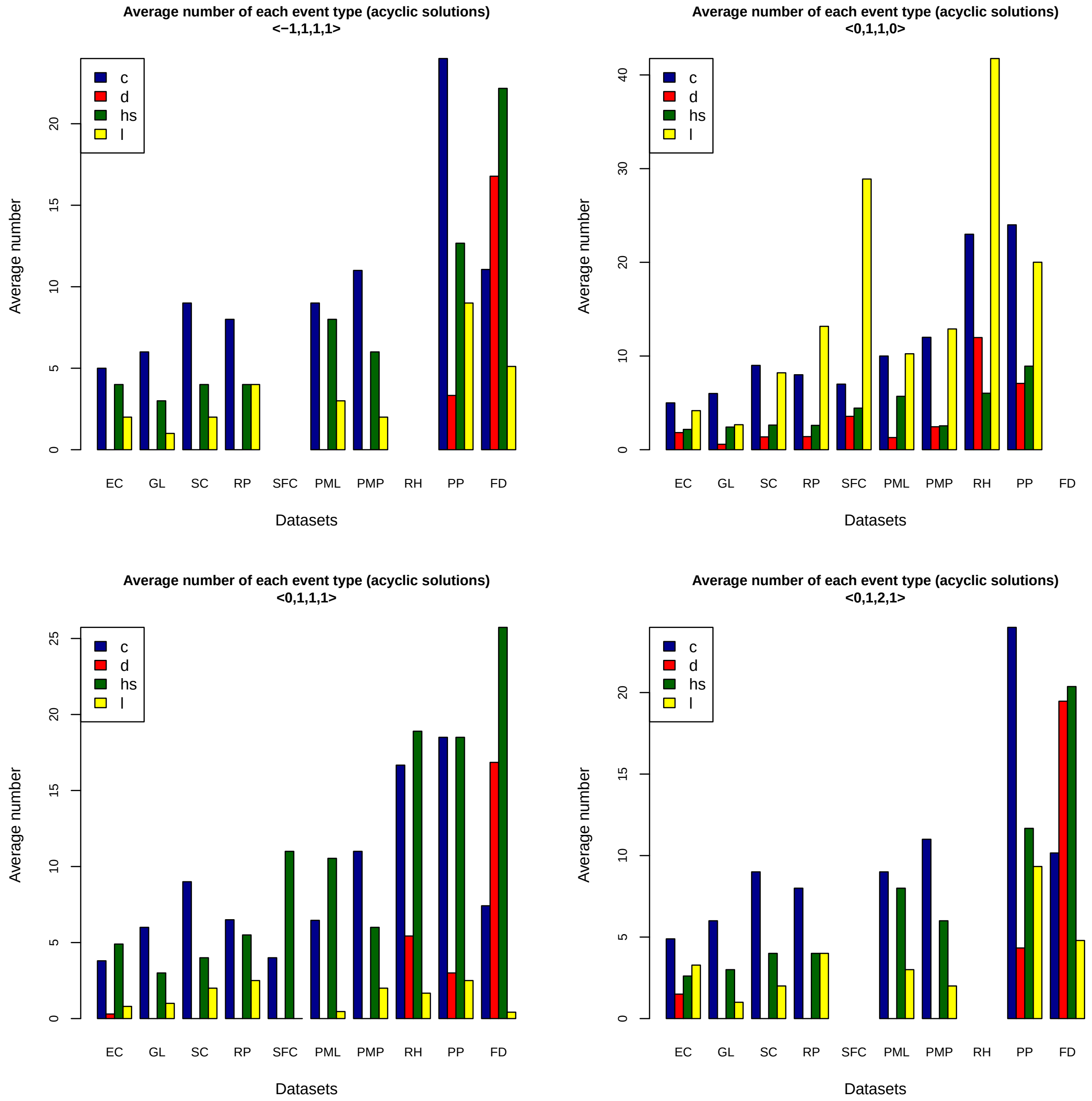

Figure 3: For each dataset we show the average number of each event in all acyclic optimal reconciliations.

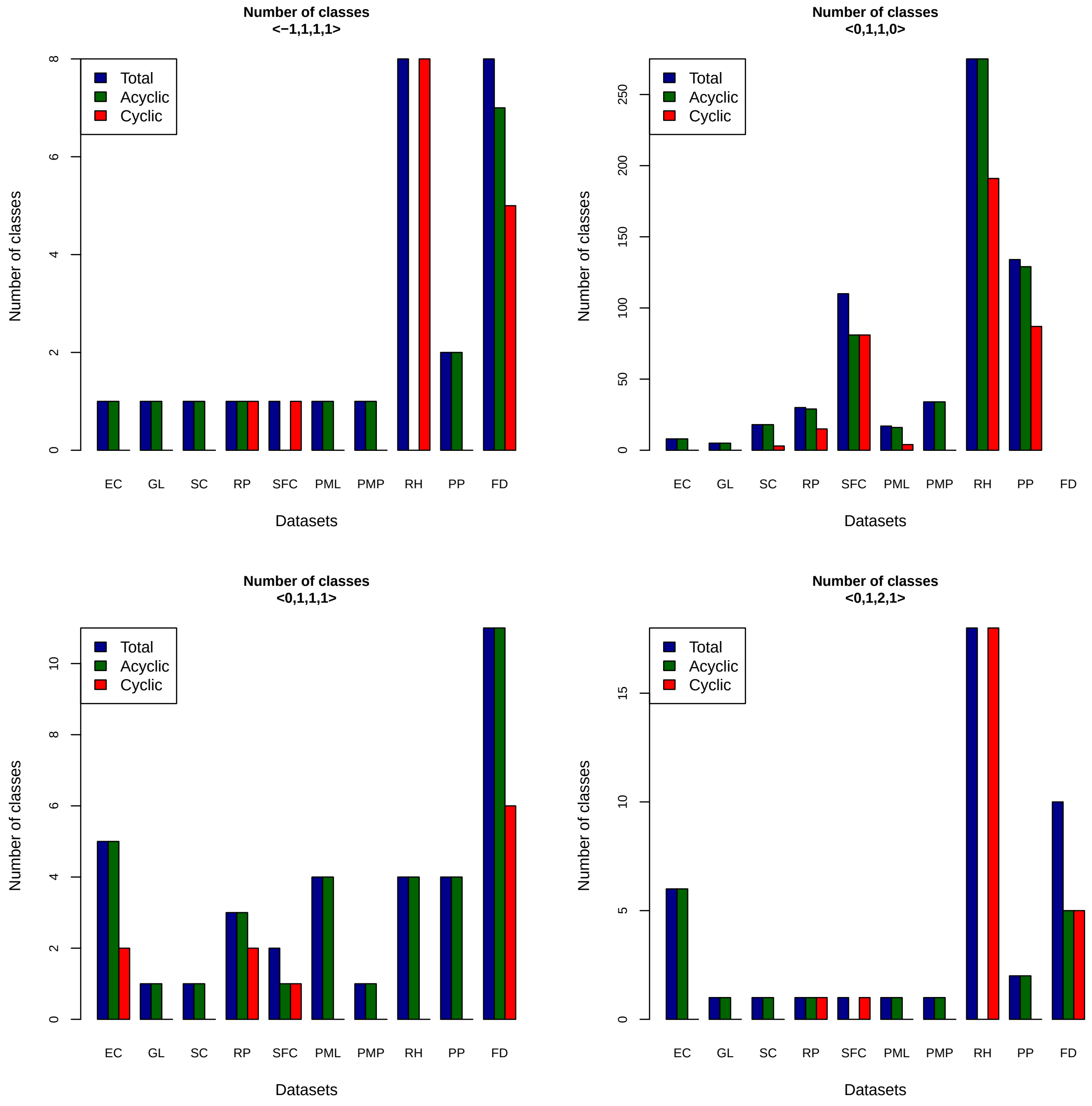

Figure 4: For each dataset we show the number of different solution classes in all optimal reconciliations, in the ones that are cyclic and also in the acyclic ones.

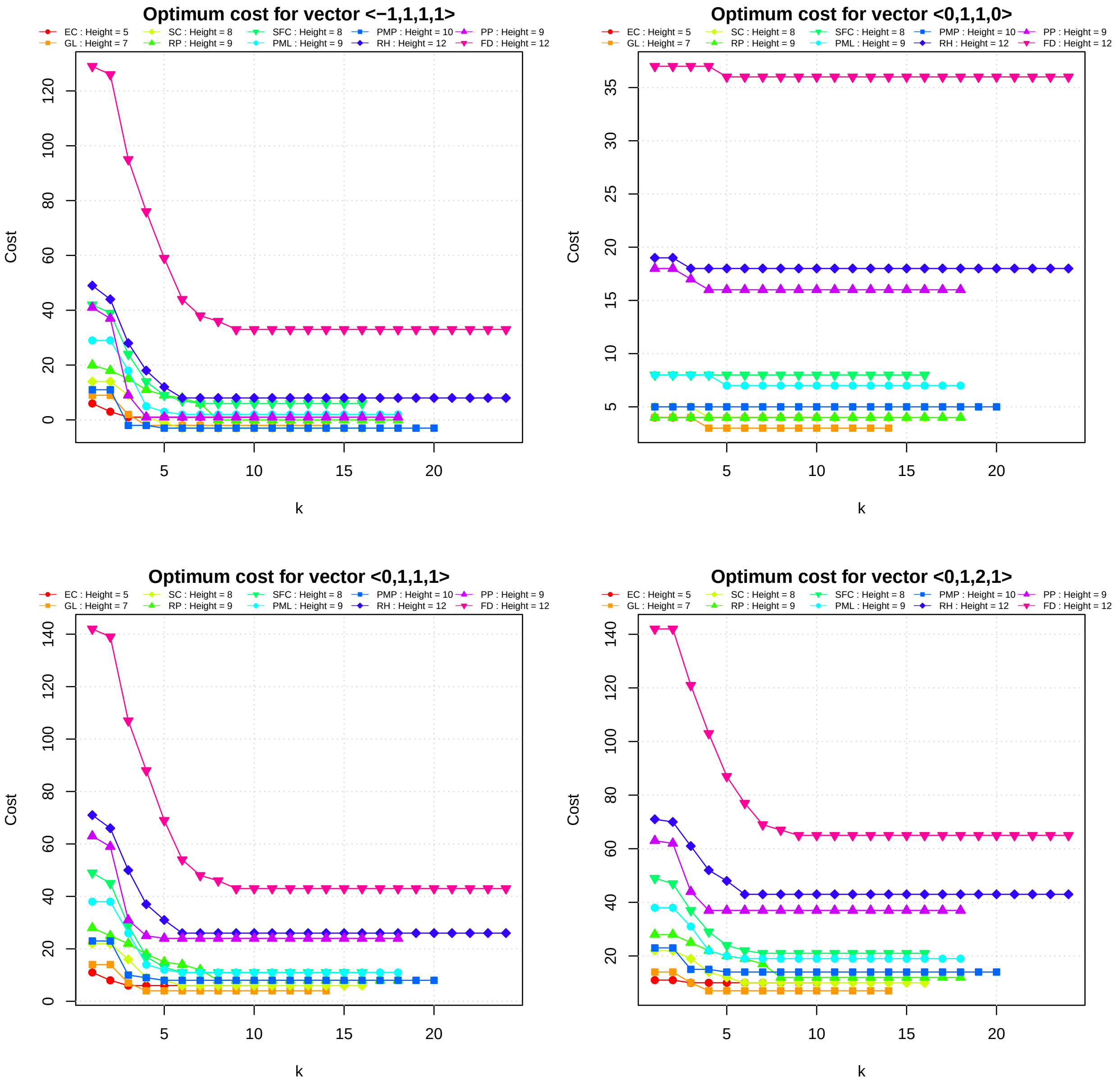

Figure 5: For all datasets we show the relation between the number of optimal solutions and the value k, of the maximum allowable distance of a switch.
